# Differences in Mood, Optimism, and Risk-Taking Behavior Between American and Chinese College Students

**DOI:** 10.3389/fpsyg.2021.781609

**Published:** 2022-01-25

**Authors:** Jiao Wang, Ruifeng Cui, Stephanie Stolarz-Fantino, Edmund Fantino, Xiaoming Liu

**Affiliations:** ^1^Center for Ideological and Political Education, Northeast Normal University, Changchun, China; ^2^Department of Psychology, University of California, San Diego, La Jolla, CA, United States; ^3^Department of Psychology, Northeast Normal University, Changchun, China

**Keywords:** mood, optimism, risk, decision-making, cultural, national

## Abstract

Mood and optimism have been demonstrated to influence risk-taking decisions; however, the literature on mood, optimism, and decision-making is mixed and conducted primarily with western samples. This study sought to address this gap in the literature by examining the impact of mood and dispositional optimism on risk-taking and whether these associations differed between undergraduate students from the United States (*N* = 141) and the People’s Republic of China (*N* = 90). Both samples completed a dispositional optimism questionnaire and an autobiographical mood induction task. They were then tasked with choosing to complete the Raven’s Advanced Progressive Matrices reasoning task on easy, medium, or hard difficulty for hypothetical money. Selecting harder difficulties was interpreted as more risk-taking due to a higher chance of failure. More positive mood and higher dispositional optimism were associated with decreased risk-taking, i.e., selecting easier puzzle difficulties, in the American sample but increased risk-taking decisions, i.e., selecting harder difficulties, in the Chinese sample (*p* < 0.05 for all). These findings suggest that the effect of mood and optimism on decision-making may differ by nationality and/or culture.

## Highlights

– Decision-making is a process everyone engages in every day.
– The identification of state and trait factors that influence the decision-making process, especially with respect to making risky decisions, has implications for improving the understanding of suboptimal decision-making and mental disorders characterized by risk-taking.
– The Affect Infusion Model posits that positive mood increases risk-taking tendencies by highlighting the positive aspects of risk-taking whereas the Mood-Maintenance Hypothesis predicts that positive mood will lead to decreased risk-taking to avoid potentially ruining a positive mood.

– The literature on the relationship between mood and risk-taking is mixed, conducted with predominantly western samples, and rarely accounts for trait factors that may underlie the decision-making process.
– This study investigates whether the influence of mood states on risk-taking decision-making may differ based on trait dispositional optimism and nationality.
– We made the case that the Chinese participants would take less risk when in a positive mood consistent with the Mood-Maintenance Hypothesis whereas the American participants would take more risk when in a positive mood consistent with the Affect Infusion Model and that the influence of mood on decision-making in both samples would be moderated by dispositional optimism.

## Introduction

“The biggest risk a person can take is to do nothing,” a quote by Robert Kiyosaki, highlights that all decisions including inaction confer a degree of risk and benefit. The identification of state and trait characteristics associated with risk-taking decisions would have implications for a better understanding of suboptimal economic decision-making and clinical disorders characterized by risk-taking. Mood states and trait optimism are two factors found to be associated with risk-taking decisions; however, this literature is mixed. Investigating how mood and optimism may interact to influence decision-making within a national/cultural context may help to reconcile and elucidate the mixed literature on the relationship among mood, optimism, and risk-taking decision-making.

Several major theoretical frameworks compete to explain the relationship between mood and risk-taking. The Affect Infusion Model is a theory that proposes that positive mood increases risk-taking tendencies by heightening the perceived positive aspects of risk-taking and minimizing the perceived negative aspects of risk-taking (Forgas, 1995). Similarly, the negative mood is hypothesized to decrease risk-taking by increasing sensitivity to the perceived negative consequences of risk-taking and decreasing sensitivity to the perceived positive consequences of risk-taking (Forgas, 1995). The Affect Infusion Model has been used to predict and explain the relationship between better mood and greater willingness to take risks/risk-taking in several past studies (e.g., Chou et al., 2007; Grable and Roszkowski, 2008).

In contrast, the Mood-Maintenance Hypothesis predicts that a positive mood will lead to decreased risk-taking as individuals will seek to avoid actions that may result in negative consequences and ruin their mood (Isen and Patrick, 1983). Similar to the Mood-Maintenance Hypothesis, the Mood Repair Hypothesis predicts that negative mood will lead to increased risk-taking as individuals seek to repair their mood (Morris and Reilly, 1987). Several studies have also provided support for the Mood-Maintenance Hypothesis and Mood Repair Hypothesis (e.g., Zhao, 2006; Juergensen et al., 2018).

The overall literature on the relationship between mood and risk-taking is mixed with some studies finding a relationship between positive mood and more risk-taking and/or negative mood and less risk-taking (e.g., Chinese sample, Chou et al., 2007; English sample, Herman et al., 2018; Italian sample, Panno et al., 2015). However, other studies have found the opposite relationship between mood and risk-taking (e.g., American sample, Buelow and Suhr, 2013) or no relationship between these two factors (e.g., Clark et al., 2001).

Dispositional optimism, which is conceptualized as the generalized expectations of a person regarding future life events across different domains (Scheier and Carver, 1985), is a personality trait that has received limited attention in the risk-taking literature. Numerous studies have found that dispositional optimism is positively correlated with positive emotions (You et al., 2009; Kapikiran, 2012) and negatively correlated with depression (Kube et al., 2018). As dispositional optimism is associated with positive mood and positive expectations regarding future outcomes, this trait may thus predispose a person toward risk-taking, in line with the Affect Infusion Model. However, this limited literature is also mixed. Some studies have found a relationship between higher dispositional optimism and greater risk-taking (e.g., German sample, Dohmen et al., 2018; American sample, Gibson and Sanbonmatsu, 2004), but other studies have found the opposite relation (e.g., Israeli sample, Barel, 2019) or no relation (e.g., Poland sample, Macko and Tyszka, 2009).

Research has found that dispositional optimism may interact with mood to influence the perceived likelihood of future positive events of an individual (Gherasim et al., 2016). This interaction may thus also influence the risk-taking tendencies of an individual. A more positive mood may heighten the preexisting risk-taking tendencies among those with high dispositional optimism whereas a negative mood may temper the risk-taking affinities of an optimist. Studying the interaction between mood and dispositional optimism may thus help to reconcile the mixed literature on the relationship among mood, dispositional optimism, and risk-taking.

Finally, the influence of culture and nationality is often overlooked in the risk-taking literature and may be contributing to the mixed findings on the relationship among mood, optimism, and risk-taking. Research indicates that individuals from western societies are more gain promotion-oriented whereas individuals from eastern societies are more loss prevention-orientated (Kurman and Hui, 2011). Western individuals with more positive moods may take greater risks for greater potential gains in line with the Affect Infusion Model whereas eastern individuals with more positive moods may take lesser risks to prevent losing and ruining their mood, consistent with the Mood-Maintenance Hypothesis. In fact, limited research suggests that the cultural background of a person may influence their risk-taking tendencies (Weber and Hsee, 2000; Kim and Park, 2010); however, no studies have investigated whether the influence among mood, dispositional optimism, and risk-taking differs by cultural or national background.

This study investigated the relationship among mood states, dispositional optimism traits, and risk-taking decisions in two separate samples of college students from the United States and China. The study aimed to draw samples from the United States and mainland China to investigate the differences in risk-taking decision-making between individuals from western individualistic and eastern collectivist cultures, respectively (Meisel et al., 2016). It was hypothesized that the Affect Infusion Model would predict risk-taking behaviors in American students whereas the Mood Maintenance Model would predict risk-taking behaviors in Chinese students. American students with more positive moods are hypothesized to take greater risks to increase the chance to gain greater reward, consistent with the Affect Infusion Model. Chinese students with more positive moods are hypothesized to take lower risks to decrease the chance for losing, consistent with the Mood-Maintenance Hypothesis. Higher optimism is hypothesized to be associated with better mood and thus more risk-taking in American students and less risk-taking in Chinese students. Mood and optimism are hypothesized to interact to predict even more risk-taking in American students and even less risk-taking in Chinese students.

The identification of state and trait factors that can influence risk-taking has implications for improving decision-making among individuals. Understanding how mood states and trait dispositional optimism relates to risk-taking can inform interventions to modify these state and trait characteristics in order to promote optimal decision-making, i.e., changing state and trait characteristics in order to increase risk-taking when it is advantageous and to decrease risk-taking when it is disadvantageous.

## Materials and Methods

### Participants

The American sample consisted of 141 college students, and the Chinese sample consisted of 90 college students. The study was conducted at a university in the Western United States and a university in Eastern China. Recruitment took place between 2011 and 2014. The study was advertised through hard copy advertisements placed around the two college campuses and through emails and word-of-mouth to students of the two colleges. The study took place over the course of a single 1-h session in person in a sound-attenuated room in a research laboratory and was carried out by trained research assistants.

Participants were included if they were 18 years or older and were proficient in English for the American sample and proficient in Mandarin for the Chinese sample. Participants were excluded if they had prior experiences with the decision-making task used in the study; 19 American and six Chinese participants were excluded. The study followed the standards of the Declaration of Helsinki and was approved by the ethics committees of the respective universities. All students provided written informed consent and received course credit for participation.

The study aimed to recruit a minimum of 100 participants per group, consistent with the statistical recommendations (Long, 1997; G*Power, Faul et al., 2007). However, the recruitment goal was barely missed for the Chinese sample, and thus, the analyses were slightly underpowered for the Chinese sample.

### Measures

*Dispositional optimism* was measured using the Revised Life Orientation Test (LOT-R; Scheier et al., 1994) for the American participants and its Chinese version (CLOT-R; Lai and Yue, 2000) for the Chinese participants. All items were rated on a 7-point Likert scale from 1 (strongly disagree) to seven (strongly agree) with a sum score that ranged from six (strongly pessimistic) to 42 (strongly optimistic). The Cronbach’s alpha was 0.77 for the American sample and 0.52 for the Chinese sample.

*Autobiographical memory recall task* was used in this study to induce a broad distribution of mood states on a positive to negative continuous spectrum (Jallais and Gilet, 2010). This method has been used to successfully induce both happy and sad mood states in numerous past research studies (e.g., Jallais and Gilet, 2010; Mills and D’Mello, 2014). Participants were randomized to a happy, sad, or neutral condition where they were instructed to recall and write about a happy experience, sad experience, or the experience of traveling to the study laboratory, respectively. The participants rated their mood states using the Brief Mood Introspection Scale (BMIS; Mayer and Gaschke, 1988). Scores were calculated based on the pleasant-unpleasant scale scoring criteria of the BMIS with higher scores indicating a more positive mood.

*Raven’s Advanced Progressive Matrices* is a non-verbal test of reasoning where individuals are provided seven geometric patterns and must complete the pattern by selecting the last missing pattern out of a choice of six to eight patterns (Raven et al., 1998). The main Raven’s task used in this study had three possible difficulty levels, namely, easy, medium, and hard, with each difficulty level set having 10 questions (Raven’s items 1–10). Participants had to choose to complete the easy, medium, or hard difficulty in order to earn 1, 2, or 4 hypothetical dollar(s)/Chinese yuan(s) per correct answer. The easy difficulty was interpreted as the low-risk choice, given a high likelihood of answering questions correctly but having low reward per correct answer. The hard difficulty was interpreted as the high-risk choice as the likelihood of answering questions correctly was low but the reward per correct answer was high.

Prior to choosing the difficulty level and completing the main Raven’s task, participants were given the opportunity to complete the Raven’s Practice Test that consisted of six questions total that were not included in the main Raven’s task. The practice set consisted of sets of two easy, two medium, and two hard questions (Raven’s items 11–12). The experimenter provided the same feedback to all participants that they answered three of the six practice questions correctly regardless of their actual performance on the practice task. This standardized deception feedback was provided so that all participants across both countries would have similar perceptions of their ability to solve Raven’s questions. Finally, American participants did not significantly differ compared to Chinese participants on the actual number of correct answers on the Raven’s Practice Test, suggesting the comparable levels of cognitive ability and achievement orientation in the two samples [American Mean Correct = 2.52, SD = 1.39; Chinese *M* = 2.79, SD = 1.37; *F*(1,229) = 0.65, *t* = −1.42, *p* = 0.16].

The Raven’s task was chosen as the indices of risk-taking for several reasons. This task, unlike many other behavioral risk-taking tasks (e.g., Balloon Task; Lejuez et al., 2002), tends to be more novel to psychology undergraduates, thus allowing for a more unbiased assessment of risk-taking. The measure also allows for the assessment of risk-taking based on individual merit under unambiguous circumstances unbiased by chance/luck and the subjective perceptions of subjects of chance/luckiness, which are potential confounds in many other risk-taking scenarios (e.g., Dohmen et al., 2011).

### Procedures

All participants completed the study in groups of two to five individuals in a sound-attenuated room. Instructions and written materials were provided in English for the American sample and Mandarin Chinese for the Chinese sample. Participants first completed the dispositional optimism questionnaire followed by the Raven’s Practice Task. They were subsequently randomly assigned to recall and write about a positive, negative, or neutral memory. Participants then chose a Raven’s difficulty level to complete to earn hypothetical money and then completed it. Finally, participants answered a demographics questionnaire. The optimism questionnaire was administered first to assess optimism unbiased by other study tasks, and the mood induction was administered and mood assessed right before the Raven’s task choice to maximize the impact of transient mood state on task choice.

### Data Analytic Plan

Analyses were conducted using SPSS 19.0 (IBM, Armonk, NY, IBM Corp.). A series of ANOVAs and chi-square tests examined nationality and gender differences among study variables. Multinomial logistic regressions were used to analyze whether the independent variables of mood states, trait dispositional optimism, and their interactions were significantly associated with the dependent variable of Raven’s difficulty choice for students from the United States and China separately. Models were rerun using different Raven’s difficulty choices as the reference group so that all pairwise comparisons were made (i.e., predicting whether easy difficulty as the reference comparison group differed as compared to the medium and hard difficulties; medium difficulty as reference group vs. easy and hard difficulty; and hard difficulty as reference group vs. easy and medium difficulty, respectively).

## Results

Descriptive characteristics are presented in Table 1. The mood induction task was successful in both the American and Chinese samples. Among American participants, average mood ratings for the happy, neutral, and sad conditions were 46.11 (SD = 7.48), 43.60 (SD = 6.48), and 41.57 (SD = 7.51), respectively [*F*(2,138) = 4.71, *p* = 0.01]. With respect to the Chinese participants, average ratings for the happy, neutral, and sad conditions were 50.16 (SD = 7.10), 46.39 (SD = 7.58), and 41.10 (SD = 7.64), respectively [*F*(2,87) = 11.61, *p* < 0.01]. Chinese participants had higher levels of dispositional optimism (*M* = 29.61, SD = 4.87) than American participants (*M* = 27.29, SD = 6.12; Table 1). No gender differences were found both for dispositional optimism levels and induced mood levels. The Chinese participants chose the higher risk hardest difficulty more often than the American participants [χ^2^(2, *N* = 231) = 9.41, *p* < 0.01; Table 2]. Among both the American and Chinese participants, men tended to choose the hardest difficulty more often than women [χ^2^(2, *N* = 141) = 6.53, *p* = 0.04; χ^2^ (2, *N* = 90) = 17.35, df = 2, *p* < 0.01; respectively; Table 2]. Gender was thus controlled for in all multinomial logistic regressions. Despite differences in difficulty choice, Chinese and American participants did not differ in Raven’s task performance or total earnings (*p* > 0.05 for all).

**TABLE 1:** Demographic characteristics.

	American Students		Chinese Students				
	*M*	SD	*M*	SD	*F* or χ^2^	df	*p*
Age	20.86	1.89	22.32	1.34	6.398	229	<0.001
% Men	29%		49%				
Mood^1^	43.76	7.36	45.87	8.28	2.021	229	0.044
Optimism^2^	27.29	6.12	29.61	4.87	3.022	229	0.003
# Raven’s Correct^3^	6.31	2.73	5.61	2.52	1.939	229	0.054
Raven’s Earnings^4^	12.32	5.78	12.54	4.96	0.305	229	0.761

*^1^Brief Mood Introspection Scale; ^2^Revised Life Orientation Test; ^3^Raven’s Advanced Progressive Matrices # correct; and ^4^Hypothetical money earned on Raven’s task.*

*F, ANOVA test; χ^2^, chi-square test.*

**TABLE 2:** Differences in decision-making between the American and Chinese participants.

	American			Chinese		
		
	Male *N* (%)	Female *N* (%)	Total *N* (%)	Male *N* (%)	Female *N* (%)	Total *N* (%)
Easy	4 (9.76)	21 (21)	25 (17.73)	3 (6.82)	9 (19.57)	12 (13.33)
Medium	25 (60.98)	66 (66)	91 (64.54)	16 (36.36)	30 (65.22)	46 (51.11)
Difficult	12 (29.27)	13 (13)	25 (17.73)	25 (56.82)	7 (15.22)	32 (35.55)
Total N	41	100	141	44	46	90

Among American participants, higher overall mood states and trait dispositional optimism were associated with an increased likelihood of choosing the higher risk option of completing the Raven’s task on hard difficulty over the medium difficulty (Table 3). Every point increase in mood and optimism was associated with 29.5% [*B* = −0.35, exp(*B*) = 0.71] and 46% [*B* = −0.62, exp(*B*) = 0.54] decreased likelihood of selecting the medium difficulty as compared to the hard difficulty, respectively. There was an interaction between mood and optimism such that American participants with a more positive mood were less likely to choose the higher risk harder difficulties if they had higher dispositional optimism [*B* = 0.01, exp(*B*) = 1.01].

**TABLE 3:** Multinomial logistic regression analysis predicting easy, medium, and difficult task choices based on dispositional optimism and affective states by country.

Raven’s decision		American			Chinese		
			
		B (SE)	*p*	OR (95% CI)	B (SE)	*p*	OR (95% CI)
Easy vs. Medium	Constant	−0.25 (5.33)	0.96		−29.00 (16.36)	0.08	
	Gender	−0.75 (0.61)	0.22	0.47 (0.14, 1.55)	−1.24 (0.83)	0.14	0.29 (0.06, 1.49)
	Optimism	−0.03 (0.21)	0.91	0.98 (0.65, 1.46)	1.11 (0.59)	0.06	3.04 (0.95, 9.66)
	Affective States	0.01 (0.12)	0.92	1.01 (0.80, 1.28)	0.82 (0.41)	0.05*	2.26 (1.01, 5.07)
	Affective States * Optimism	−0.001 (0.01)	0.89	1.00 (1.00, 1.00)	−0.03 (0.02)	0.04*	0.97 (0.94, 1.00)
Medium vs. Difficult	Constant	16.91 (8.01)	0.04*		−2.52 (7.07)	0.72	
	Gender	−0.91 (0.48)	0.06	0.40 (0.16, 1.03)	−2.02 (0.57)	0.01**	0.13 (0.04, 0.41)
	Optimism	−0.62 (0.29)	0.03*	0.54 (0.30, 0.95)	0.11 (0.24)	0.65	1.11 (0.69, 1.79)
	Affective States	−0.35 (0.18)	0.05*	0.71 (0.50, 1.00)	0.12 (0.17)	0.48	1.13 (0.81, 1.57)
	Affective States * Optimism	0.01 (0.01)	0.03*	1.01 (1.00, 1.03)	−0.003 (0.01)	0.54	1.00 (0.99, 1.01)
Easy vs. Difficult	Constant	16.66 (8.93)	0.06		−31.51 (16.47)	0.06	
	Gender	−1.66 (0.70)	0.02*	0.19 (0.05, 0.75)	−3.26 (0.92)	0.01**	0.04 (0.01, 0.23)
	Optimism	−0.65 (0.33)	0.05*	0.52 (0.27, 1.00)	1.22 (0.59)	0.04*	3.38 (1.06, 10.81)
	Affective States	−0.33 (0.20)	0.09	0.72 (0.49, 1.06)	0.94 (0.42)	0.03*	2.55 (1.12, 5.79)
	Affective States * Optimism	0.01 (0.01)	0.07	1.01 (1.00, 1.03)	−0.04 (0.02)	0.02*	0.97 (0.94, 1.00)

*Affective states were measured by the Brief Mood Introspection Scale; and optimism was measured by the Revised Life Orientation Test in the American sample and the Chinese Revised Life Orientation Test in the Chinese sample.*

*p < 0.05 and **p < 0.01.

With respect to the Chinese participants, the main effect of and the interaction between mood and optimism on decision-making in the Raven’s task remained significant, but the directions were reversed (Table 3). Higher mood and optimism were associated with an increased likelihood of choosing the lower risk easy Raven’s difficulty level over the hard difficulty. Every point increase in mood and optimism was associated with 155% [*B* = 0.94, exp(*B*) = 2.55] and 238% [*B* = 1.22, exp(*B*) = 3.38] increased likelihood of selecting the easy difficulty compared to hard difficulty, respectively. The interaction between mood and optimism indicated that Chinese participants with a more positive mood were less likely to choose the lower risk easier difficulty if they had higher dispositional optimism [*B* = −0.04, exp(*B*) = 0.97].

## Discussion

This study is the first to investigate the relationship between mood states, trait dispositional optimism, and their interaction on risk-taking among undergraduate students from the United States and undergraduates from China. Compared with American participants, more Chinese participants chose to complete the Raven’s task on the hard difficulty, which entailed a higher risk of failure, i.e., answering questions incorrectly, but also higher reward per correct answer. The finding of greater risk-taking on the Raven’s task in the Chinese sample as compared to the American sample is consistent with the past literature on risk-taking differences between these two nationalities (e.g., risk-taking measured by gambling task, Meisel et al., 2016). These findings support the cushion hypothesis that states that individuals from socially collectivist cultures, e.g., China, may take greater financial risks because their social networks would help “cushion” any losses incurred (Weber and Hsee, 2000). Acting as part of a group on a risk-taking task, i.e., cooperating with others, has been shown to increase risk-taking (Liu et al., 2021). The Chinese participants in this study may have viewed their actions as actions undertaken as part of their social network/in cooperation with their social network and thus demonstrated higher risk-taking compared to their American counterparts who likely viewed their actions as individual actions (Liu et al., 2021). Additionally, cooperation typically conveys a degree of risk and thus cooperation may also be conceptualized as a type of risk-taking (Engel and Zhurakhovska, 2016). Consequently, the greater risk-taking in the Chinese participants may also be indicative of greater affinity toward cooperation. In fact, past research suggests that individuals from collectivistic cultures may be more willing to cooperate than individuals from individualistic cultures (Leung and Au, 2010). Positive mood was associated with higher likelihood of choosing the harder and thus more risky Raven’s difficulty in the American sample and a higher likelihood of choosing the easier and thus less risky Raven’s difficulty in the Chinese sample, consistent with our hypotheses. The risk-taking decisions of the American sample were in line with the Affect Infusion Model which posits that a positive mood heightens the perceptions of an individual of positive aspects of risk-taking, thus increasing risk-taking (Forgas, 1995). Conversely, findings in the Chinese sample support the Mood-Maintenance Hypothesis which posits that a positive mood may decrease the risk-taking of individuals as they may avoid taking risks to avoid potentially ruining their positive mood (Isen and Patrick, 1983).

As hypothesized, American participants with higher levels of dispositional optimism tended to choose the more risky hard Raven’s difficulty whereas Chinese participants with higher optimism tended to choose the less risky easy Raven’s difficulty. Findings are consistent with prior literature indicating that Americans with greater optimism may make riskier choices and take fewer preventative steps to avoid adverse outcomes (Gibson and Sanbonmatsu, 2004). With respect to the Chinese students, Chinese society places high emphasis and pressure on success in academic tasks among students, and poor performance is often met with censure and viewed as a negative reflection on the individual, family, and community (Li, 2001). In this context, high dispositional optimism may have arisen and been maintained among students through a positive cycle of engaging in easy academic tasks, e.g., Raven’s easy difficulty, resulting in frequent successes, thus leading to increased/maintained high levels of optimism.

In summary, a more positive mood and more optimism may influence American participants to take more risks and may influence Chinese participants to take less risks. As cooperation conveys a degree of risk/requires a degree of risk-taking, future studies may investigate whether more positive mood and optimism are associated with more affinity toward cooperation in Americans and less affinity toward cooperation in Chinese.

Finally, mood interacted with optimism to influence risk-taking in both the American and Chinese samples. The association between more optimism and more risk-taking in American students was weaker among those with a more positive mood, and the association between more optimism and less risk-taking in Chinese students was weaker among those with a more positive mood. This was in contrast to our hypothesis that mood would strengthen the relationship between optimism and risk-taking. However, these results are similar to those from a study by Gherasim et al. (2016), which found that a positive affect weakened the relationship between dispositional optimism and judgments about the likelihood of future positive events. Positive moods have been shown to increase the consideration of alternative solutions and outcomes (Politis and Houtz, 2015). It may be that individuals in a positive mood are more aware of other courses of action and their potential outcomes, thus weakening the affinity toward the course of action favored by their optimistic dispositional trait. The present findings suggest that the buffering effects of positive mood states on the relationship between trait dispositional optimism and risk-taking decision-making have cross-national stability.

Much of the studies on risk-taking and factors associated with risk-taking have been conducted with western samples/in western countries. Our findings suggest that factors associated with greater risk-taking in participants from a western country, e.g., positive mood, may have the opposite influence on participants from an eastern country and highlight the need to investigate the construct of risk-taking within national and cultural frameworks. Future studies may also investigate whether modifying mood and optimism will lead to changes in risk-taking behaviors. Interventions aiming to decrease maladaptive risking in American samples may consider reigning in high mood and optimism whereas interventions to decrease maladaptive risk-taking in Chinese samples may consider promoting high mood and optimism. This study has several limitations that influence the interpretation and generalizability of the findings. The Cronbach’s alpha of CLOT-R was low, which may have negatively impacted the measurement of trait optimism among the Chinese sample. Eastern cultures may differ from western cultures in the conceptualization/meaning of optimism, which may have contributed to the suboptimal Cronbach’s alpha of the translated CLOT-R measure (Lai and Yue, 2000). The study used a convenience sample of college students who typically tend to be young and from high socioeconomic backgrounds, thus limiting generalizability. The hypothetical money Raven’s decision task used in this study is novel and has not been used to measure risk-taking in previous studies, and thus, the findings may also not generalize to other analog measures of risk-taking, especially those using real money rewards. Future studies may seek to replicate and extend present findings using other behavioral measures of risk-taking, e.g., Balloon Task (Lejuez et al., 2002) and Lottery Task (Dohmen et al., 2011), and using real money incentives. Modest-sized participant samples were recruited from only two countries, and the study did not assess the cultural affiliations of participants and identifies which hinders drawing inferences regarding the influence of culture. Findings would benefit from replication in larger more diverse samples of non-college student participants from multiple countries using a multimodal approach to assessing risk-taking decision-making.

Notwithstanding these limitations, this study was the first to investigate the impact of mood states and trait dispositional optimism on risk-taking decision-making across both American and Chinese samples. Findings indicated that higher trait dispositional optimism moderated the relationship between more positive mood states and greater risk-taking behavior in the American sample. In contrast, higher optimism moderated the relationship between more positive mood states and decreased risk-taking in the Chinese sample. These findings indicate that mood and optimism independently as well as jointly interact to influence risk-taking decisions, and the influence of mood and optimism on risk-taking differs by the national background of an individual.

## Data Availability Statement

The raw data supporting the conclusion of this article will be made available by the authors, without undue reservation.

## Ethics Statement

The studies involving human participants were reviewed and approved by Northeast Normal University Institutional Review Board and University of California San Diego Institutional Review Board. The patients/participants provided their written informed consent to participate in this study.

## Author Contributions

JW, RC, SS-F, EF, and XL contributed to the conception and design of the study. JW and RC wrote the first draft of the manuscript and recruited the participants. JW, RC, and XL performed the statistical analyses. All authors contributed to the manuscript revision, as well as read and approved the submitted version.

## Conflict of Interest

The authors declare that the research was conducted in the absence of any commercial or financial relationships that could be construed as a potential conflict of interest.

## Publisher’s Note

All claims expressed in this article are solely those of the authors and do not necessarily represent those of their affiliated organizations, or those of the publisher, the editors and the reviewers. Any product that may be evaluated in this article, or claim that may be made by its manufacturer, is not guaranteed or endorsed by the publisher.
